# Transcriptomic analysis between self- and cross-pollinated pistils of tea plants (*Camellia sinensis*)

**DOI:** 10.1186/s12864-018-4674-1

**Published:** 2018-04-25

**Authors:** Qingping Ma, Changsong Chen, Zhongping Zeng, Zhongwei Zou, Huan Li, Qiongqiong Zhou, Xuan Chen, Kang Sun, Xinghui Li

**Affiliations:** 1 Tea Research Institute, Nanjing Agricultural University, Nanjing, 210095 China; 2 Tea Research Institute, Fujian Academy of Agricultural Sciences, Ningde, 355015 China; 3 Department of Plant Science, University of Manitoba, Winnipeg, R3T 2N2 Canada

**Keywords:** Self-incompatibility, Ion components, Pollen tube growth, Transcriptome

## Abstract

**Background:**

Self-incompatibility (SI) is a major barrier that obstructs the breeding process in most horticultural plants including tea plants (*Camellia sinensis*). The aim of this study was to elucidate the molecular mechanism of SI in tea plants through a high throughput transcriptome analysis.

**Results:**

In this study, the transcriptomes of self- and cross-pollinated pistils of two tea cultivars ‘Fudingdabai’ and ‘Yulv’ were compared to elucidate the SI mechanism of tea plants. In addition, the ion components and pollen tube growth in self- and cross-pollinated pistils were investigated. Our results revealed that both cultivars had similar pollen activities and cross-pollination could promote the pollen tube growth. In tea pistils, the highest ion content was potassium (K^+^), followed by calcium (Ca^2+^), magnesium (Mg^2+^) and phosphorus (P^5+^). Ca^2+^ content increased after self-pollination but decreased after cross-pollination, while K^+^ showed reverse trend with Ca^2+^. A total of 990 and 3 common differentially expressed genes (DEGs) were identified in un-pollinated vs. pollinated pistils and self- vs. cross-pollinated groups after 48 h, respectively. Function annotation indicated that three genes encoding UDP-glycosyltransferase 74B1 (UGT74B1), Mitochondrial calcium uniporter protein 2 (MCU2) and G-type lectin *S*-receptor-like serine/threonine-protein kinase (G-type RLK) might play important roles during SI process in tea plants.

**Conclusion:**

Ca^2+^ and K^+^ are important signal for SI in tea plants, and three genes including UGT74B1, MCU2 and G-type RLK play essential roles during SI signal transduction.

**Electronic supplementary material:**

The online version of this article (10.1186/s12864-018-4674-1) contains supplementary material, which is available to authorized users.

## Background

Self-incompatibility (SI) is a common phenomenon in plant reproduction system, which prevents self-fertilization in flowering plants. There are two classical known mechanisms for SI, namely, homomorphic gametophytic self-incompatibility (GSI) and homomorphic sporophytic self-incompatibility (SSI). In GSI system, the pollen incompatibility (haploid male gametophyte) is controlled by the *S* allele, pollen and pistils bearing the same *S* allele trigger an incompatible reaction [1]. While in SSI system, incompatibility is determined by both *S* alleles of the (diploid-sporophyte) pollen parents [2].

GSI has been found in many plant species, such as Solanaceae [3, 4] and Rosaceae [5–7], while SSI is typically found in Brassicaceae [8]. Both GSI and SSI have male or female determinate conditions which are regulated by different prominent genes [9]. In GSI system, *S* locus-encoded F-box (SLF/SFB) proteins control the pollen recognition of S-RNase based SI [10–12]. In SSI systems, *S*-locus receptor kinase (*SRK*) gene and *S*-locus cysteine-rich protein (SCR)/*S*-locus protein-11 (SP11) function as a receptor-ligand pair to recognize self-pollens at the surface of stigma epidermal papilla cells [13]. The *SRK* is a membrane-spanning receptor protein in stigma containing an extracellular domain (*S*-domain) for recognition of *SP11*, a transmembrane domain, and an intracellular serine/threonine kinase domain [14]. S-locus glycoprotein gene (*SLG*) and *SRK* exhibit series characteristics which are associated with the female determinant of SSI in *Brassica* [15]. The *S* domain of *SRK* is highly similar to the *SLG*, which is the first *S*-locus gene to be identified and a soluble glycoprotein secreted to the stigma surface [16, 17]. Besides, the pollen coat protein *SCR/ SP11* controls pollen determinant of SSI in *Brassica* [8, 18], and many *SP11*, *SRK*, and *SLG* alleles were inherited together to term different S haplotypes.

Self-incompatibility mechanism remains unclear in tea plant. Previous studies suggested that tea plant SI might be in late-acting self-incompatibility system (LSI), in that self-pollinated pollen tubes elongated through the style but failed in fertilization [19, 20]. This has made it almost impossible to obtain fruits in self-pollinated tea plants (*Camellia sinensis*); thus, breeding process in tea plant is not encouraged. LSI is a novel SI system in plants, but the molecular mechanism of this system is still unclear. Recently, Zhang et al. [21] found that tea plant SI might be categorized to GSI through transcriptome analysis. Therefore, the SI mechanism in tea plants is still controversial and needs further exploration.

To understand the mechanism of SI in tea plant, the ion components and pollen tube growth in self- and cross-pollinated pistils were investigated. Furthermore, the transcriptome of self- and cross-pollinated pistils of two tea cultivars ‘Fudingdabai’ and ‘Yulv’ was compared to figure out the DEGs which may be involved in SI of tea plant. ‘Fudingdabai’ is a national superior clone and cultivated widely in China because of its good quality, high yield, and excellent stress resistance, while ‘Yulv’ is a high-quality cultivar selected from the hybrid offsprings of ‘Yabukita’. Both cultivars are self-incompatible and show high fruiting rates after cross-pollination. This study will provide reference for understanding SI mechanism of tea plant.

### Methods

### Plant materials and treatments

**Fig. 1:**
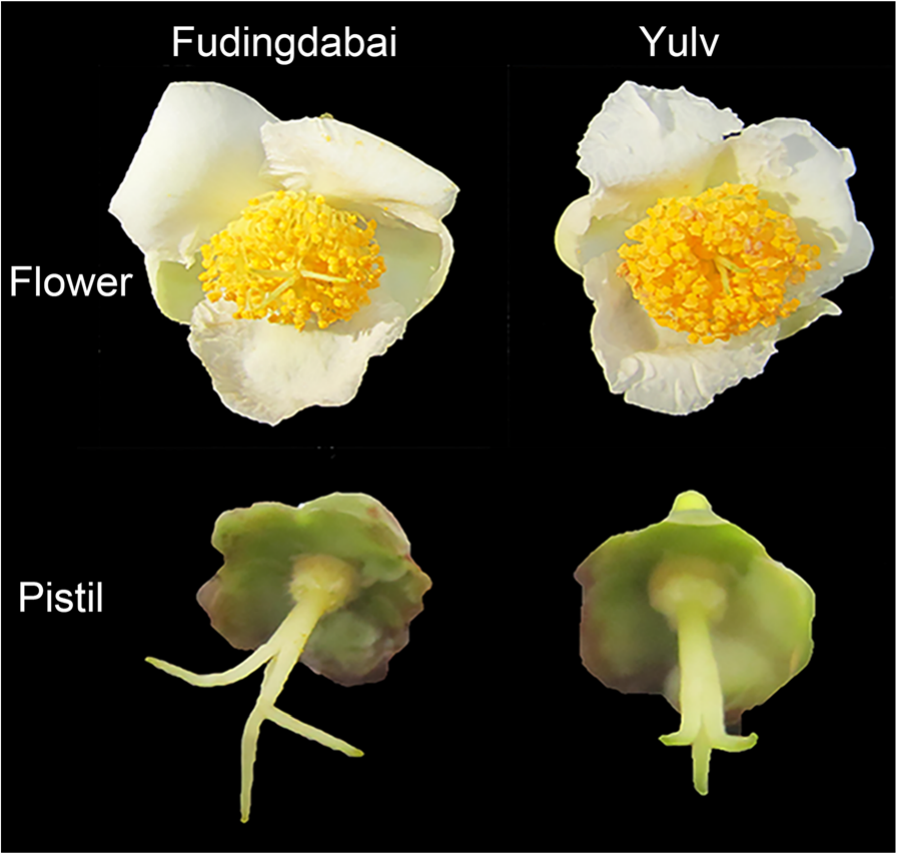
Morphology of flowers from ‘Fudingdabai’ and ‘Yulv’ cultivars

Two ten-year-old tea cultivars, namely, *C. sinensis* cv. Fudingdabai and *C. sinensis* cv. Yulv, cultivated in tea germplasm repository of Tea Research Institute of Fujian Academy of Agricultural Sciences were used in this study. Flowers from both of the two tea cultivars have three petals and trifid stigmas. The stigmas of ‘Fudingdabai’ divided at the base but ‘Yulv’ at the upper part (Fig. 1).

**Fig. 2:**
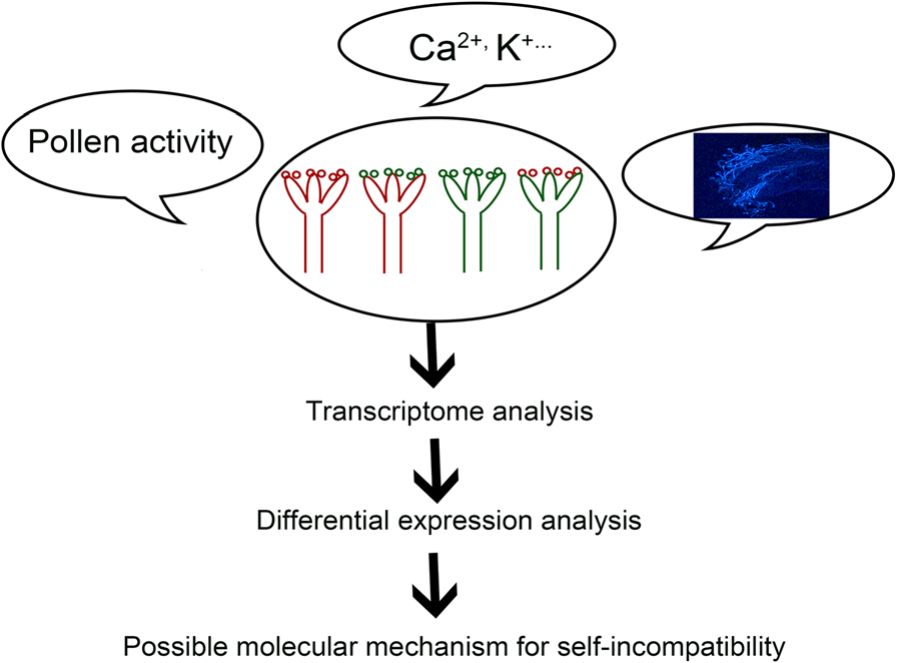
The flow diagram of the experiment design. Red and green indicate pollination combination of the two tea cultivars in this study. The color dots mean pollens from the corresponding cultivars

Flower buds of the two tea cultivars were harvested at 4:00 pm for pollens collection. Besides, the remaining flower buds of two cultivars were emasculated and used for artificial pollination next morning. A total of four pollination combinations were conducted: ‘Fudingdabai’ (♂) × ‘Fudingdabai’ (♀), ‘Yulv’ (♂) × ‘Yulv’ (♀), ‘Fudingdabai’ (♂) × ‘Yulv’ (♀), and Yulv’ (♂) × ‘Fudingdabai’ (♀), as shown in Fig. 2. The un-pollinated and pollinated pistils at 8, 24, 48 and 72 h were picked from each combination and frozen quickly in liquid nitrogen and stored at − 80 °C for RNA extraction. Three biological replicates were conducted with at least five pistils for each replicate.

### Pollen culture in vitro

Pollen culture medium was prepared with the following substances: 0.59 g MES, 0.02 g H_3_BO_3_, 0.05 g Ca(NO_3_)·4H_2_O, 5 g sucrose and 5 g polyethylene glycol 4000 (PEG 4000), diluted with distilled water to 100 mL. Pollens were cultured in the medium in the dark and observed by Olympus light microscope (Olympus, Tokyo, Japan). Pollen germination rate and mean pollen tube length at 1, 2, and 4 h were calculated based on eight visual fields.

### Fluorescence activity of pollen tube

Fresh un-pollinated and pollinated styles were fixed in FAA fixative buffer (5 mL formalin, 6 mL acetic acid, and 89 mL 50% ethanol) for 24 h [19]. The styles were washed with deionized water and then softened by 2 M NaOH overnight. The softened styles were stained by 0.1% aniline blue solution dissolved with 0.15 M K_2_HPO_4_. Finally, the styles were observed under Leica DM6B fluorescence microscope (Leica, Bannockburn, USA) after 15 min staining. At least five styles were observed for each sample.

### Ion components of pistils

Un-pollinated and pollinated styles were dried at 80 °C for 4 h. A total of 0.1 g dried samples (at least 15 pistils) were ground and digested in 5 ml nitric acid by ETHOS One high performance microwave digestion system (Milestone, Bergamo, Italy) for 1 h. The digested samples were diluted with nitric acid to 25 mL and analyzed by inductively coupled plasma-optical emission spectrometer (PerkinElmer Optima 2100DV, Massachusetts, USA). A total of nine ions were detected, including potassium (K^+^), calcium (Ca^2+^), magnesium (Mg^2+^), phosphorus (P^5+^), zinc (Zn^2+^), boron (B^3+^), Ferrous (Fe^2+^), aluminium (Al^3+^), and manganese (Mn^2+^). The contents of the ions were quantified by establishing standard curve.

### RNA extraction, library construction and sequencing

Total RNA was extracted using Plant RNA extraction kit (Bioteke, China) according to the manual. RNA quality and concentration were assessed by 1% agarose gels, Qubit®2.0 Fluorometer (Invitrogen, Carlsbad, USA), and Agilent Bioanalyzer 2100 system (Agilent, Palo Alto, USA). A total of 3 μg RNA per sample was used for sequencing libraries preparation by NEBNext®Ultra™ RNA Library Prep Kit for Illumina® (NEB, USA) following manufacturer’s instructions and library quality was assessed on the Agilent Bioanalyzer 2100 system (Agilent Technologies, Palo Alto, CA). The clustering of samples was performed using TruSeq PE Cluster Kit v3-cBot-HS (Illumina) according to the manufacturer’s instructions. Finally, sequencing analysis was carried out with an Illumina Hiseq 2500 platform to generate pair-end reads.

### Genome alignment and gene annotation

Raw data of fastq format were processed, and then were cleaned by trimming the adapter sequences, ploy-A containing reads and low quality reads. The clean reads were aligned to the reference genome (http://www.plantkingdomgdb.com/tea_tree/) by TopHat2 using the default parameters [22]. The mapped reads were assembled into possible transcripts by Cufflinks [23]. The unannotated transcripts were annotated by BLAST [24] based on the following databases: NR (NCBI non-redundant protein sequences) [25], COG (Clusters of Orthologous Groups of proteins) [26], Swiss-Prot (A manually annotated and reviewed protein sequence database) [27], KEGG (Kyoto Encyclopedia of Genes and Genomes) and GO (Gene Ontology) [28].

### Identification of differentially expressed genes

Fragments per Kilobase of transcript per Million mapped reads (FPKM) estimates produced by RNA-Seq by Cuffquant and Cuffnorm of Cufflinks was used to evaluate the expression of transcripts [29]. The transcriptome comparisons of un-pollinated vs. pollinated groups and self-pollinated vs. cross-pollinated groups were conducted to find the differentially expressed genes (DEGs). Differential expression analyses were performed using the DESeq R package 1.10.1 [30], which provides statistical routines to determine DEGs based on a negative binomial distribution model. The *P* values were adjusted by the Benjamini and Hochberg’s approach for controlling the false discovery rate [31]. False discovery rate < 0.01 and fold change > 2 was considered to be significantly differentially expressed. Pearson’s Correlation Coefficient was used to evaluate the correlation of biological repeats [30].

### Quantitative real time PCR verification

The first-strand cDNA was synthesized from 1 μg of total RNA by using the RevertAid™ First Strand cDNA Synthesis Kit (Thermo Scientific, MA, USA) according to the manual. Quantitative real time PCR (QRT-PCR) was performed using SYBR Premix EX Taq (Takara, Japan) on Roche LightCycler® 480II (Switzerland) as instruction specified. The qRT-PCR primers (Table 1) were designed by using Primer Premier 5.0 (Premier Biosoft International, Palo Alto, CA). The *GAPDH* (GenBank: GE651107) from tea plant was used as the reference gene. All of the PCR reactions were conducted in triplicate and the average expression values were calculated. The relative expression level of each gene was calculated with the 2^-ΔΔCT^ method [32].

**Table 1:** The primers used for qRT-PCR verification

ID	Forward (5′-3′)	Reverse (5′-3′)
CSA006398	GGCGTATCCAACAATCTTATCG	CCAAACCCAATCATCATCCA
CSA005891	GAACGTGTGTTGGTCATTGAT	CATAAATTGTCTACTGGCGAG
CSA028406	GAGATTCAGTTGTCGCTTTG	AGAGCCACCATTTCATTAGC
CSA024717	CCACTGCCACTTGTCGTTGTT	GAGTTTGCCACCGTGAATTCG
CSA002728	GTCGTTCCACTGGCTTCCTAC	GGCAGTAGTTGTTCATAGAGA
CSA026098	GGCTCCCTCTTTCTTTATATG	CCACCATCAATTTCTCCCTTG
CSA024379	TCCCATCATTAGCCTGCCAAC	ATCCCATCTCAGCCCATAAC
*GAPDH*	TTGGCATCGTTGAGGGTCT	CAGTGGGAACACGGAAAGC

### Statistical analysis

The statistical analysis was conducted using Excel 2016 and GraphPad Prism 5.0 (San Diego, USA). The significance analysis of difference between two samples was evaluated with Student t-test and multiple comparisons were analyzed using One-way ANOVA and *P* < 0.05 was considered to be statistically significant. The results were displayed as mean ± standard deviation.

## Results

### Pollen germination in vitro and fluorescence of pollen tubes

In order to evaluate the pollen vitality, pollen germination rate and tube growth between ‘Fudingdabai’ and ‘Yulv’ were assessed and compared. As shown in Additional file 1: Figure S1A, the pollen appearance between two cultivars has no significant difference. Pollen germination rate and mean length of pollen tubes of the two cultivars were similar and increased gradually with prolongation of the growth time (Additional file 1: Figure S1B).

The fluorescence of pollen tube was observed to examine the growth of pollens in pistils (Additional file 2: Figure S2). After 8 h self-fertilization of ‘Fudingdabai’, the pollens germinated at stigma but no fluorescence was seen in styles. After 24 h, a few pollen tubes entered styles and the fluorescence on the base of styles was observed firstly after 48 h. Pollen tubes of ‘Yulv’ (♂) × ‘Fudingdabai’ (♀) cross-fertilized pistils showed higher growth rate than ‘Fudingdabai’ self-fertilization. The pollen tubes arrived at the base of styles after 24 and 8 h in self-fertilized ‘Yulv’ pistils and ‘Fudingdabai’ (♂) × ‘Yulv’ (♀) cross-fertilized pistils, respectively. Taken these results together, pollens from other cultivars would grow faster in pistils than that from themselves. This result was similar to that by Zhang et al. [21]. In addition, reciprocal cross-pollination showed that the pollen tube growth was slower when ‘Fudingdabai’ was used as maternal parent. However, pollen tubes in all of the self- and cross-pollinated samples reached the base of styles after 48 h.

### Ion components in self- and cross-fertilized pistils

**Fig. 3:**
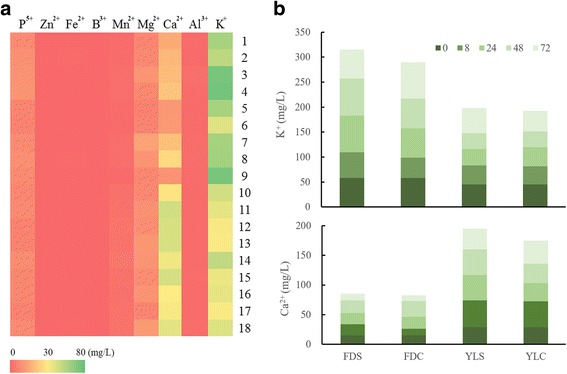
Ion components in self- and cross-pollinated pistils of tea plants. **a 1** un-pollinated ‘Fudingdabai’ pistils; **2–5** self-pollinated ‘Fudingdabai’ pistils at 8 h, 24 h, 48 h and 72 h; **6–9** ‘Yulv’(♂) × ‘Fudingdabai’(♀) at 8 h, 24 h, 48 h and 72 h; **10** un-pollinated ‘Yulv’ pistils; **11–14** self-pollinated ‘Yulv’ pistils at 8 h, 24 h, 48 h and 72 h; **15–18** ‘Yulv’(♀) × ‘Fudingdabai’(♂) at 8 h, 24 h, 48 h and 72 h. **b** The Ca^2+^ and K^+^ content during self- and cross-pollinated pistils

Ion components, especially Ca^2+^, are an indicator of self-incompatibility [33]. In tea pistils, the highest level of ion component observed in the tea pistil was K^+^, followed by Ca^2+^, Mg^2+^ and P^5+^ in sequence (Fig. 3a). Pistils of ‘Fudingdabai’ contained more K^+^ but less Ca^2+^ than those in ‘Yulv’. Ca^2+^ content in self-pollinated pistils of ‘Yulv’ (YLS) was higher than that in cross-pollinated pistils of ‘Yulv’ (YLC), but no apparent difference between self- (FDS) and cross-pollinated pistils of ‘Fudingdabai’ (FDC). In FDS pistils, the K^+^ content was higher than in FDC pistils, suggesting that Ca^2+^ and K^+^ may be involved in potential signal transduction in SI.

### Transcriptome assembly and function annotation

A total of 18 samples were sequenced and 122.75 Gb clean data were obtained. The percentages of clean reads having a base quality greater or equal than Q30 were above 85.01% indicating that the data produced by sequencing are of high quality. The clean reads from the 18 samples showed alignment ratios between 47.77% and 54.96% (SRA accession: SRP110788, Table 2). Based on the alignment with the reference genome of tea, 8136 unannotated genes were found, and 6621 of these genes were annotated after BLAST, with 1481 unigenes in COG database, 3465 in GO database, 2301 in KEGG database, 4285 in Swissprot database and 6588 in Nr database.

**Table 2:** The alignment of transcriptomic reads on genome

Sample	Total Reads	Mapped Reads (%)	Unique Mapped Reads	Multiple Map Reads
FD0–1	41,191,716	21,483,151 (52.15%)	49.14%	3.01%
FD0–2	47,556,326	22,717,253 (47.77%)	43.74%	4.03%
FD0–3	42,082,490	22,243,075 (52.86%)	49.41%	3.44%
FDS48–1	42,900,034	22,929,028 (53.45%)	49.59%	3.86%
FDS48–2	47,034,306	23,852,770 (50.71%)	46.94%	3.77%
FDS48–3	45,609,388	23,605,185 (51.76%)	48.43%	3.33%
FDC48–1	48,440,032	25,869,526 (53.41%)	50.72%	2.68%
FDC48–2	41,896,840	21,354,194 (50.97%)	47.74%	3.23%
FDC48–3	49,373,892	25,641,271 (51.93%)	49.57%	2.37%
YL0–1	46,464,662	23,481,565 (50.54%)	47.86%	2.68%
YL0–2	43,549,168	23,651,982 (54.31%)	52.27%	2.04%
YL0–3	41,549,084	21,639,699 (52.08%)	49.26%	2.82%
YLS48–1	53,246,958	26,180,870 (49.17%)	46.35%	2.82%
YLS48–2	50,605,784	27,367,119 (54.08%)	51.17%	2.91%
YLS48–3	40,743,908	21,133,266 (51.87%)	45.29%	6.57%
YLC48–1	45,900,260	25,068,461 (54.62%)	51.87%	2.74%
YLC48–2	53,049,730	29,157,364 (54.96%)	52.29%	2.67%
YLC48–3	47,106,846	24,086,169 (51.13%)	48.49%	2.64%

### Differentially expressed genes analysis

**Fig. 4:**
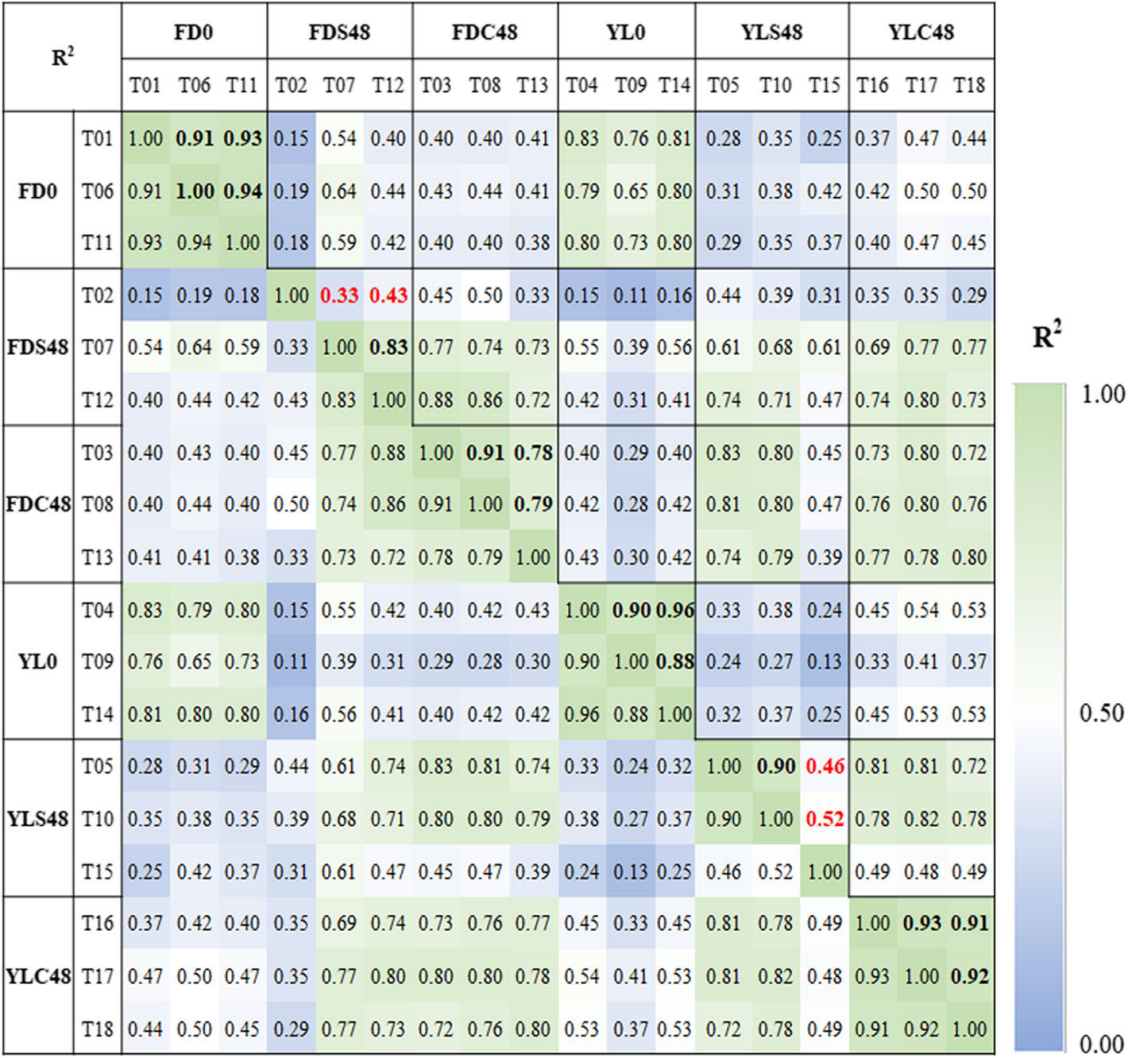
Correlation analysis of the samples for differential expression analysis. Different sample numbers represent un-pollinated ‘Fudingdabai’ pistils (FD0), self-pollinated ‘Fudingdabai’ pistils at 48 h (FDS48), ‘Yulv’(♂) × ‘Fudingdabai’(♀) at 48 h (FDC48), un-pollinated ‘Yulv’ pistils (YL0), self-pollinated ‘Yulv’ pistils at 48 h (YLS48) and ‘Yulv’(♀) × ‘Fudingdabai’(♂) at 48 h (YLC48), respectively. Bold values are R^2^ for replicates of each sample

Correlation analysis showed that T02 of self-pollinated ‘Fudingdabai’ at 48 h (FDS48) revealed low correlation to other two FDS48 samples (T07 and T12) with R^2^ of 0.33 and 0.43, respectively. T15 of self-pollinated ‘Yulv’ pistil sample at 48 h (YLS48) deviated from other two replicates (T05 and T10) with R^2^ of 0.46 and 0.52, respectively. These two samples (T02 and T15) were therefore removed in further DEG analysis. All replicates of remaining samples showed high correlation (Fig. 4).

**Fig. 5:**
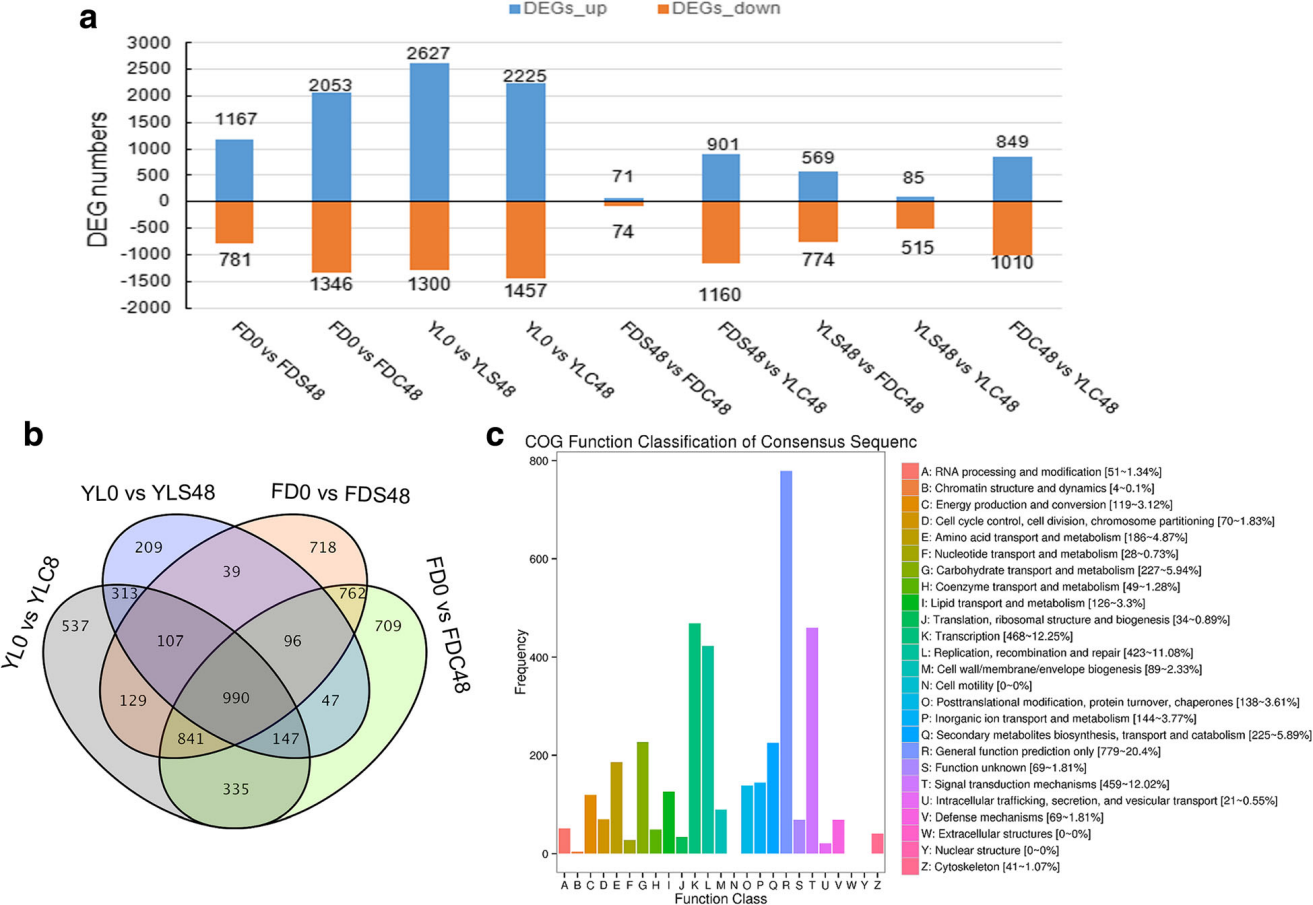
Differentially expressed genes identified in different comparisons. **a** The number of DEGs found in different comparisons. **b, c** Venn diagram and COG annotation for DEGs between un-pollinated and pollinated groups

A total of 1948, 3399, 3927, 3682, 145, 2061, 1343, 600 and 1859 genes were found to be differentially expressed between each of un-pollinated ‘Fudingdabai’ pistils (FD0) vs. FDS48, FD0 vs. cross-pollinated ‘Fudingdabai’ (♀) pistils after 48 h (FDC48), un-pollinated ‘Yulv’ pistils (YL0) vs. YLS48, YL0 vs. cross-pollinated ‘Yulv’ (♀) pistils after 48 h (YLC48), FDS48 vs. FDC48, FDS48 vs. YLC48, YLS48 vs. FDC48, YLS48 vs. YLC48 and FDC48 vs. YLC48, respectively (Fig. 5a). By comparing the pollinated groups with un-pollinated groups, 990 common DEGs were found (Fig. 5b). COG classification of these DEGs showed that ‘General function prediction only’ enriched most of DEGs, followed by ‘Transcription’, ‘Signal transduction mechanisms’, ‘Replication, recombination and repair’ and ‘Secondary metabolites biosynthesis, transport and catabolism’ (Fig. 5c). GO enrichment analysis revealed that metabolic process in biological process, cell part in cellular component and catalytic activity in molecular function enriched the most DEGs (Additional file 3: Figure S3).

### Differentially expressed genes between self- and cross-pollinated groups

**Fig. 6:**
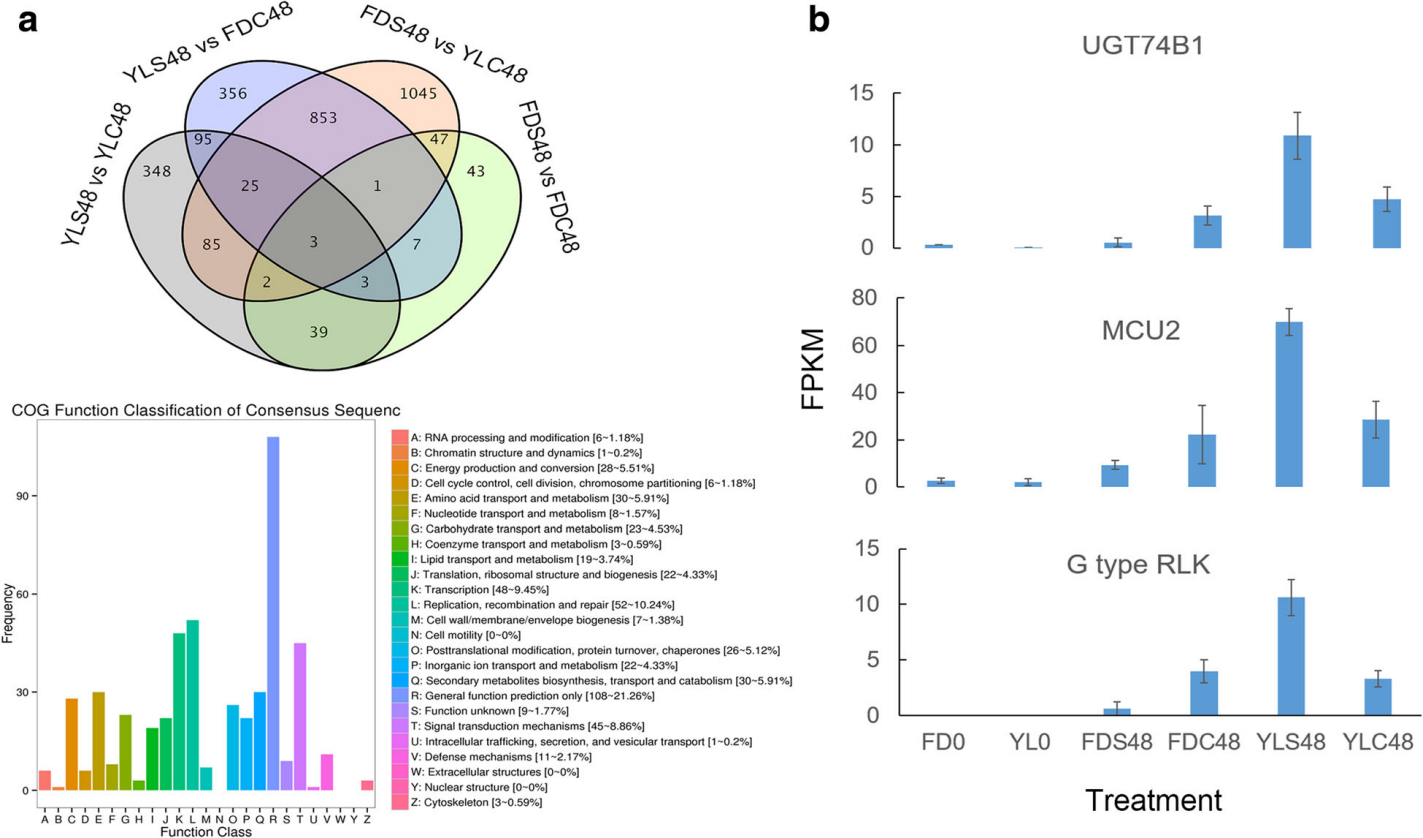
DEGs identified between self- and cross-pollinated groups. **a** Venn diagram for DEGs between self- and cross-pollinated groups. **b** The expression of the three common DEGs between self- and cross-pollinated groups

In comparison of self- and cross-pollinated groups, only three common DEGs were found (Fig. 6a). The 1160 common DEGs identified at least in two comparisons were therefore considered in further analysis. COG function classification revealed that ‘General function prediction only’ contained the most common DEGs followed by ‘Replication, recombination and repair’, ‘Transcription’ and ‘Signal transduction’. This result shows that cross-fertilization caused a series of responses in transcriptional level. In addition, the three common DEGs in all comparisons were UDP-glycosyltransferase 74B1 (UGT74B1, CSA001819), Mitochondrial calcium uniporter protein 2 (MCU2, CSA014152) and G-type lectin S-receptor-like serine/threonine-protein kinase RLK1 (G-lecRLK, Camellia_sinensis_newGene_13508). These genes showed similar expression patterns in un-pollinated and pollinated pistils. They also expressed at same levels during reciprocal cross-pollinations, but adversely expressed during self-pollinations of the two cultivars (Fig. 6b). Function annotation found that G-lecRLK was functioned on ‘Signal transduction mechanisms’. MCU2 worked on ‘Energy production and conversion’ and ‘Carbohydrate transport and metabolism’. Finally, UGT74B1 was annotated to ‘General function prediction only’.

**Fig. 7:**
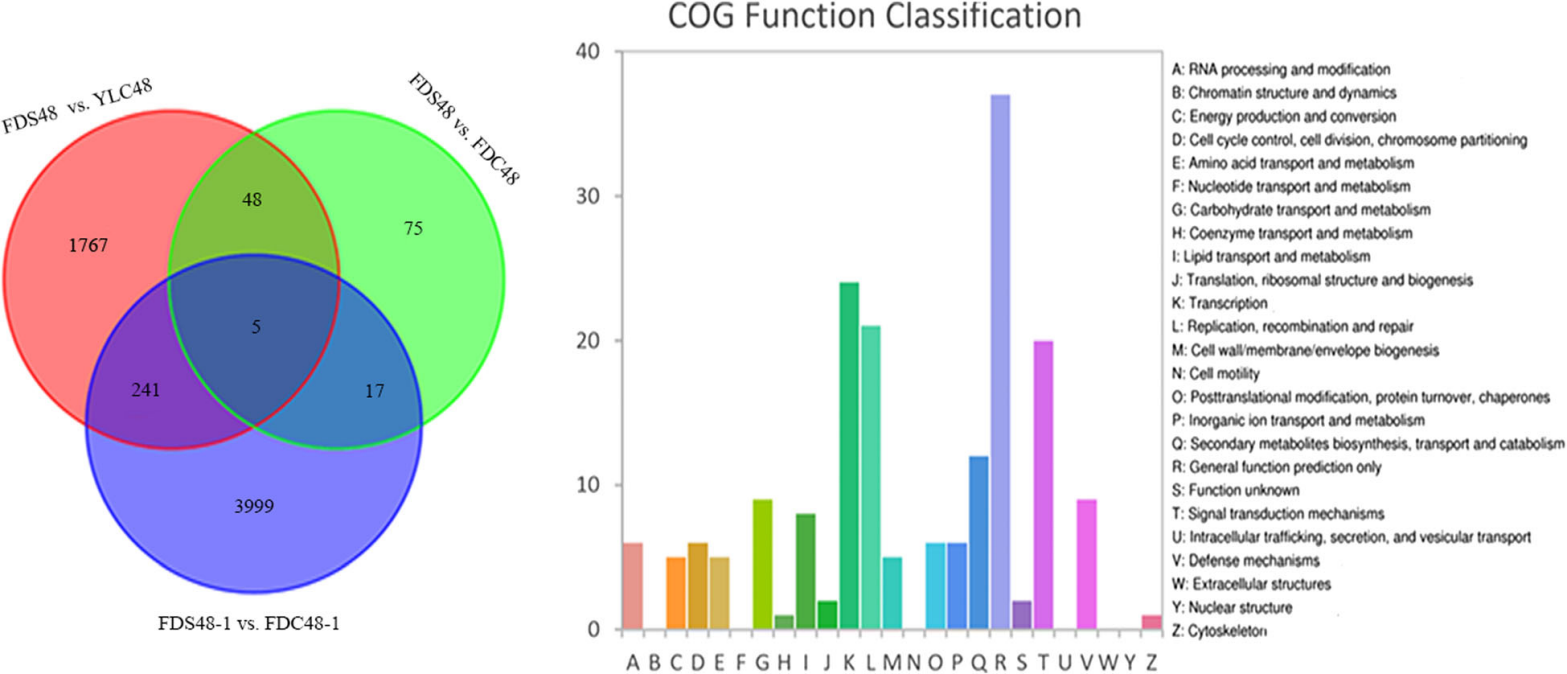
Venn diagram and COG function classification for DEGs identified between self- and cross-pollinated groups. FDS48–1 and FDC48–1 are self- and cross pollinated samples from the study by Zhang et al.

In order to compare our results to the previous study [21], the data from self-pollinated (FDS48–1, SRR3290055) and cross-pollinated ‘Fudingdabai’ samples (FDC48–1, SRR3290084) at 48 h were downloaded and re-analyzed based on genome of tea plants. A total of 4262 DEGs were identified between FDS48–1 and FDC48–1 comparison. According to the large number of DEGs, we suggested that ‘Fudingdabai’ should be used as the paternal parent in the study of Zhang et al. [21]. As shown in Fig. 7, in comparison of self- and cross-pollinated groups (FDS48 vs. FDC48, FDS48 vs. YLC48 and FDS48–1 vs. FDC48–1), only five common DEGs were filtered. Therefore, the common DEGs identified at least in two comparisons were concerned. COG function classification revealed similar result to the four groups comparisons in our study (Fig. 7). Except for ‘general function prediction only’, the classes of ‘Replication, recombination and repair’, ‘Transcription’ and ‘Signal transduction’ enriched most of the DEGs. In addition, G-lecRLK, MCU2 and UGT74B1 were found in these common DEGs, suggesting that these DEGs played vital roles during SI process. In the five overlapping genes, MCU2 and UGT74B1 were found. G-lecRLK was only expressed in the comparisons in our study because it was annotated by database blast but not genome mapping.

### DEGs between reciprocal cross-pollinations

**Fig. 8:**
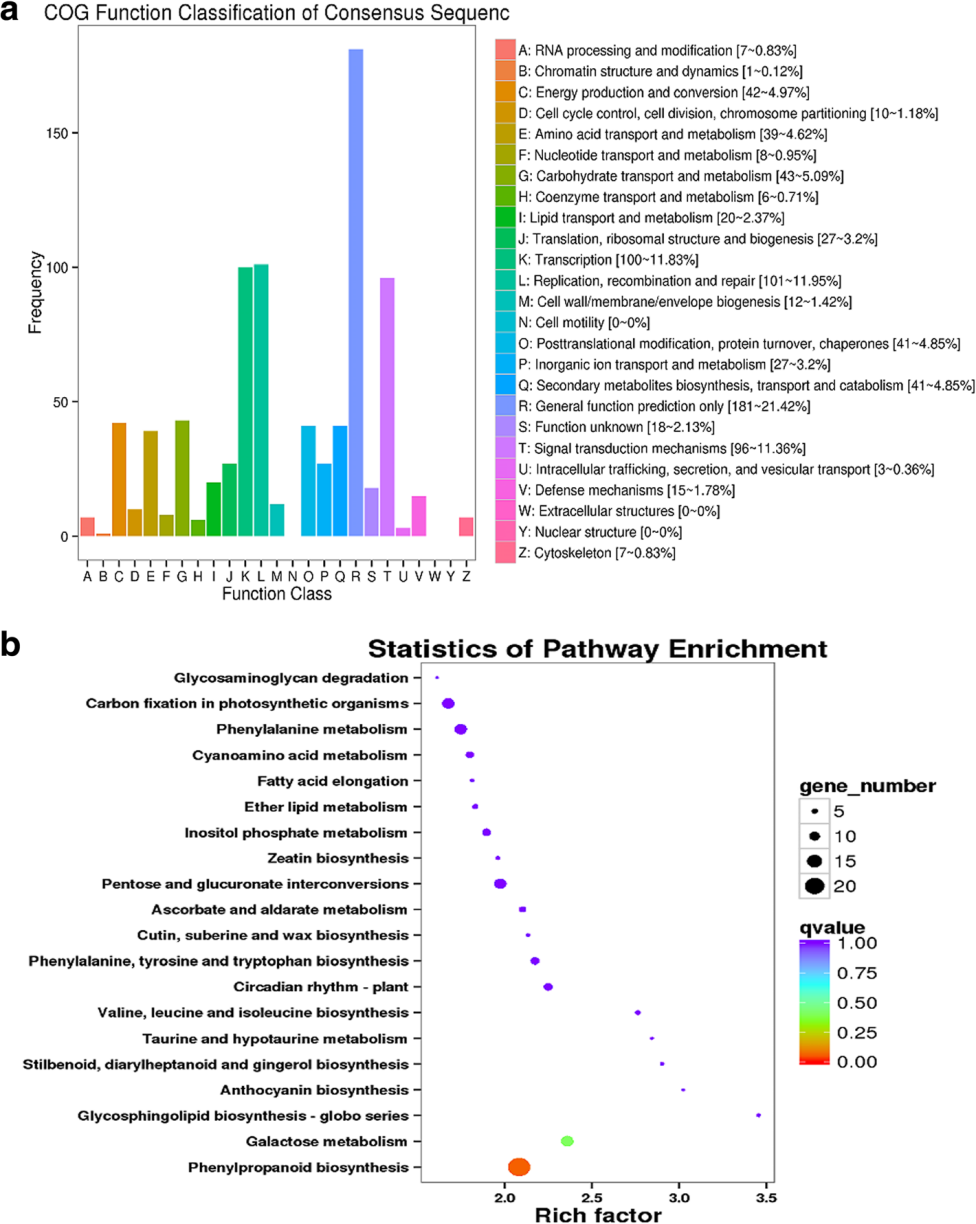
The DEGs between reciprocal cross-pollinations. **a** COG function classification of DEGs between reciprocal cross-pollinations. **b** KEGG enrichment analysis of DEGs between reciprocal cross-pollinations

In the present study, 1859 DEGs were identified in FDC48 vs. YLC48 comparison. Except for ‘General function prediction only’, most of these DEGs were involved in ‘Transcription’ and ‘Replication, recombination and repair’ (Fig. 8a). KEGG pathway enrichment analysis showed that ‘Galactose metabolism’ possessed the highest rich factor and ‘Phenylpropanoid biosynthesis’ had the most DEGs (Fig. 8b). This result suggests different responses between reciprocal cross-pollinations in tea cultivars.

### Verification of differentially expressed genes

**Fig. 9:**
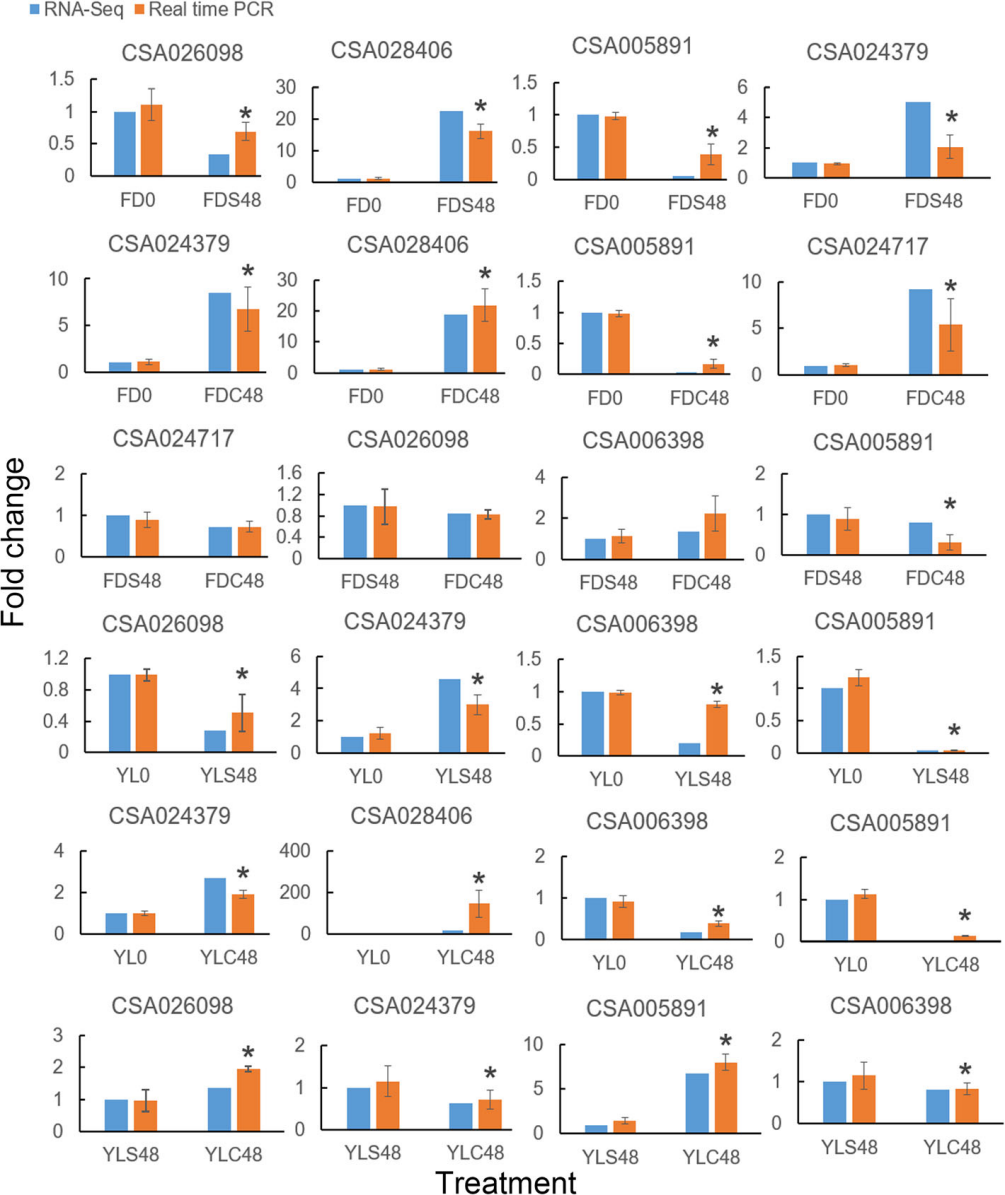
The qRT-PCR verification for RNA-Seq analysis. * means different significantly (*P* < 0.05)

In order to verify the reliability of RNA-Seq data, eight DEGs were selected for qRT-PCR analysis. As shown in Fig. 9, most of the DEGs showed similar expression trend compared to the RNA-Seq analysis. Therefore, the RNA-Seq analysis is credible.

## Discussion

Self-incompatibility is a common phenomenon in angiosperm. In order to understand the SI mechanism of tea plants, we studied the ion components and pollen growth in self- and cross-pollinated pistils from two tea cultivars. The results showed that pollen tubes grew faster in cross-pollinated pistils than those in self-pollinated pistils. Furthermore, Ca^2+^ in pistils increased after self-pollinations but decreased after cross-pollinations. In addition, comparative transcriptome analysis showed that *G-type LecRLK*, *UGT74B1* and *MCU2* genes might contribute the SI signal transduction mechanism in tea plant.

### Signal transduction during self-incompatibility in tea plants

Self-incompatibility is genetically regulated by a multi-allelic *S*-locus which links pollen and pistil *S*-determinants and resulting in self-recognition. Interactions between pollen and pistil in the same haplotype triggered a SI response, which inhibits pollen tube growth and leads to failure of fertilization [34]. During SI process, a series of signal changes occurred in plants. The earliest identified physiological event caused by SI recognition is the increase of Ca^2+^ in incompatible pollen tubes or stigma papilla cells [35, 36]. In the present study, Ca^2+^ changes suggest a potential correlation between pollen tube growth and Ca^2+^ content. Furthermore, the opposing trend of Ca^2+^ ion content of the two cultivars between self- and cross-pollinated pistils reveals that Ca^2+^ may be an important signal for SI in tea plants.

In the present study, a DEG *MCU2* was identified between self- and cross-pollination, which undertook the mitochondrial Ca^2+^ uptake [37]. In animals, Ca^2+^ uptake could regulate the mitochondrial energy production that is a stimulation of sperm-induced Ca^2+^ release [38]. There is evidence that the Ca^2+^ uptake also occurred at fertilization in mammalian eggs [39]. Inhibition of the mitochondrial function also disrupted the sperm-induced Ca^2+^ oscillatory pattern and intracellular Ca^2+^ homeostasis, and resulted in low developmental competence in mammals [40]. Unlike in animals, the functional mechanism of MCU in tea plants has been less studies, and; therefore, needs verification except for Ca^2+^, K^+^ is also sensitive to SI. In *Papaver rhoeas*, conductance of some monovalent cations, such as K^+^ and NH_4_^+^ were also stimulated by SI [41]. Interestingly, content of K^+^ changes was opposite to Ca^2+^ after pollination (Fig. 3). We can therefore propose that SI activates a nonspecific ion channel in tea plants.

### Role of self-incompatibility related genes in tea plants

LecRLK family has been classified to three subfamilies: L-type, G-type and C-type LecRLKs. This classification is supported by the structure analysis of these proteins. L-type LecRLK contains a legume lectin-like extracellular domain, and G-type LecRLK has a α-mannose binding bulb lectin domain, while C-type LecRLKs are characterized due to the presence of calcium-dependent carbohydrate-binding domain [42]. G-type LecRLKs were historically known as SRKs, since they hold the D-mannose binding lectin (B_lectin) and catalytic domain of the serine/threonine kinases.

*SRK* genes have a *S* domain which is responsible for SI in *Brassicaceae* [15, 43]. Recently, these genes were also reported to confer abiotic stress tolerance and delay dark-induced leaf senescence in rice [44]. Here, we screened a similar *SRK* gene (Camellia_sinensis_newGene_13508) from tea plant which differently expressed between self- and cross-pollinated pistils and might contribute to the signal transduction of SI in tea plant. In general, the *SRK* genes function in SI through the diversity of *S* domain. Therefore, the *S* domain of the *SRK* gene should be identified in different tea cultivars to explore the role of *SRK* on SI process of tea.

The previous studies have identified a LSI or an ovarian sterility (OS) type controlling self-incompatibility in tea plants [19, 20]. The same phenomenon was also observed in our study. However, the molecular mechanism of this SI system remained unclear until Zhang et al. [21, 45] proposed a gametophytic SI mechanism based on S-RNase control in tea plant. Unexpectedly, S-RNase gene was not found in tea plant in the present study, but three DEGs were identified in comparison between self- and cross-pollinated pistils: *G-type LecRLK*, *MCU2* and *UGT74B1*. Pollen tube reception, the crosstalk between the male and female gametophytes when pollen tubes arrive at the synergid cells of the ovule in flowering plants, mutation of *TURAN*(*TUN*) and *EVAN*(*EVN*) genes led to overgrowth of the pollen tubes inside the female gametophyte and inhibited the rupture of pollen tubes. *TUN* encodes a UGT superfamily protein and is required for pollen tube growth and integrity by affecting the stability of the pollen-specific *FERONIA RLKs* [46, 47]. In this work, whether the UGT74B1 and G-type RLK genes work together on fertilization in tea plant remains unknown. Nevertheless, we can suggest that both of the genes may codetermine the SI mechanism in tea plant.

It is difficult to explain that *G-type LecRLK*, *MCU2* and *UGT74B1* showed so different expression patterns between self-pollinations. It may be due to the variety difference of tea plants. More cultivars should be adopted to detect the expression of these genes in self- and cross-pollinations to interpret their roles in SI. Besides, function analysis through transgenic test to clarify the mechanism of these two genes in SI will be a good way in the future if more studies can be conducted to overcome the barriers in tea plant transformation. Our study suggests a distinctive mode of action of SI in tea, and the results therein provide new guidance and reference for exploration of SI mechanism in tea.

## Conclusion

The present study revealed that cross-pollination could promote the growth of pollens in styles and Ca^2+^ and K^+^ are involved in signal transduction in SI process of tea plants, and also *G-type LecRLK* and *UGT74B1* may function together in controlling SI in tea plants. However, the specific role of these genes in SI process needs further identification. Our study will help understand the SI mechanism of tea plant further.

## Additional files

Additional file 1: **Figure S1.** Pollen appearance and activity between ‘Fudingdabai’ and ‘Yulv’ in vitro. **A** Pollen germination and phenotype of ‘Fudingdabai’ and ‘Yulv’. **B** Pollen germination rate and average length of pollen tubes of ‘Fudingdabai’ and ‘Yulv’. (TIF 1137 kb)

Additional file 2: **Figure S2.** Fluorescence of pollen tubes in self- and cross-pollinated pistils of tea plants at 48 h. “Top” and “Base” means the stigma and the base of the style of tea flower, respectively. Arrows indicate the pollen tubes with fluoresce. (TIF 4517 kb)

Additional file 3: **Figure S3.** Gene Ontology enrich analysis of DEGs between unpollinated and pollinated samples. (TIF 755 kb)

## Abbreviations

DEGs: Differentially expressed genes
GSI: Gametophytic self-incompatibility
G-type RLK: G-type lectin *S*-receptor-like serine/threonine-protein kinase
LSI: Late-acting self-incompatibility system
MCU2: Mitochondrial calcium uniporter protein 2
SI: Self-incompatibility
SLG: S-Locus glycoprotein gene
SRK: *S*-Locus receptor kinase
SSI: Sporophytic self-incompatibility
UGT74B1: UDP-glycosyltransferase 74B1
