# Supplementary figures and images for: Transcriptomic analysis between self- and cross-pollinated pistils of tea plants (Camellia sinensis)
Source: BMC Genomics. 2018 Apr 25;19:289. doi: 10.1186/s12864-018-4674-1 (PMC5918555; doi:10.1186/s12864-018-4674-1)

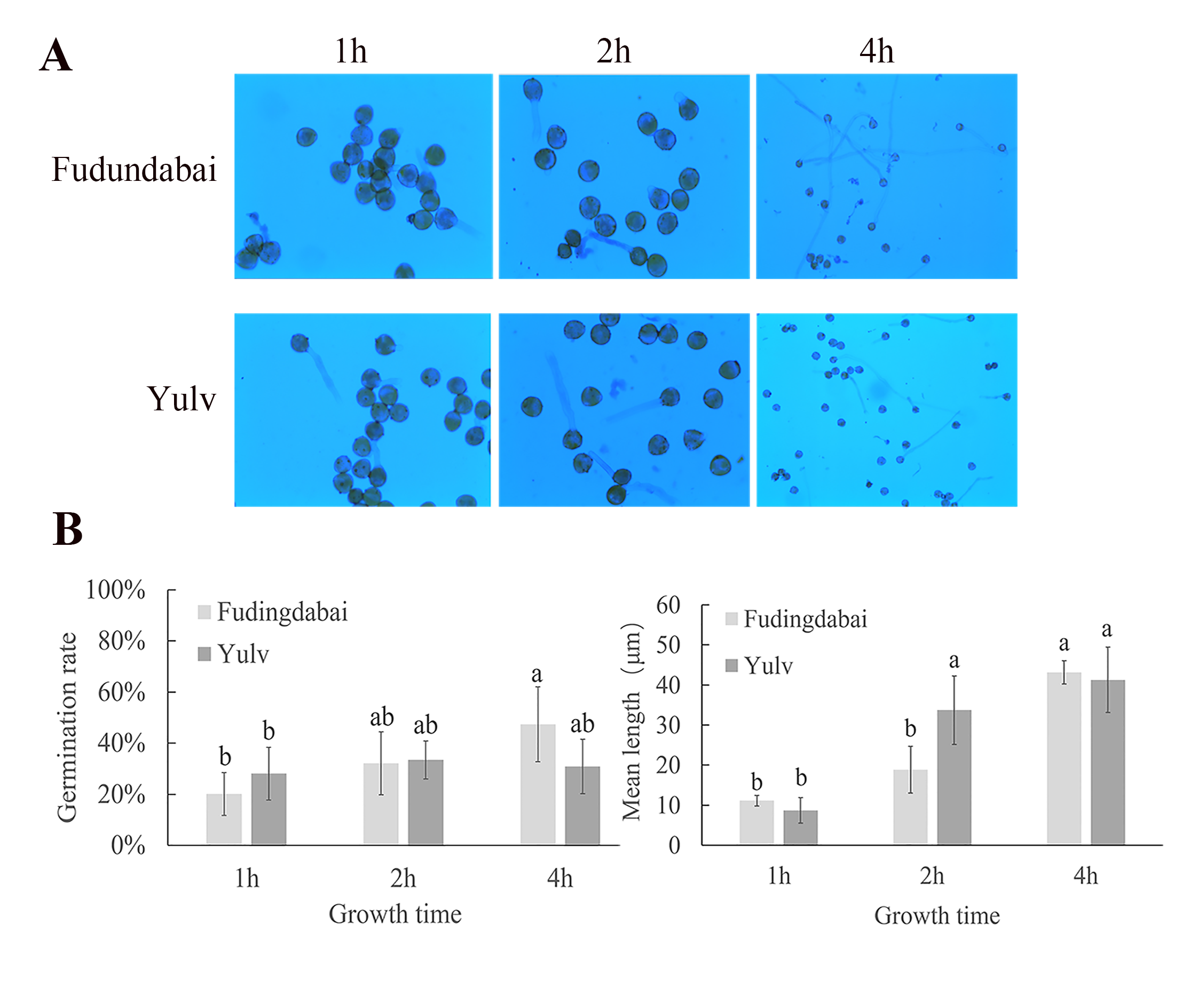

Supplement: Supplementary file 1 — Figure S1. Pollen appearance and activity between ‘Fudingdabai’ and ‘Yulv’ in vitro. A Pollen germination and phenotype of ‘Fudingdabai’ and ‘Yulv’. B Pollen germination rate and average length of pollen tubes of ‘Fudingdabai’ and ‘Yulv’. (TIF 1137 kb) [file 12864_2018_4674_MOESM1_ESM.tif]

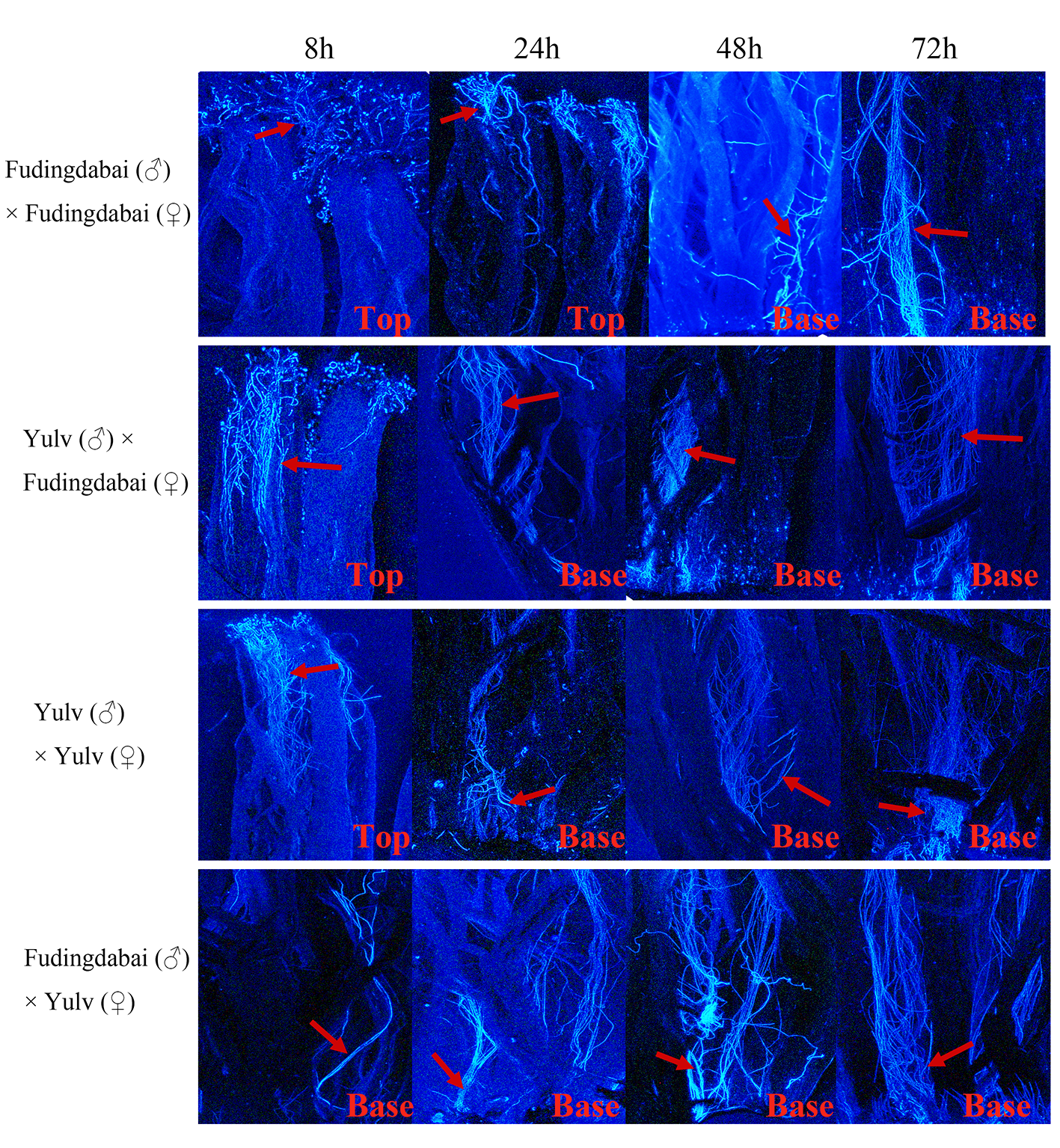

Supplement: Supplementary file 2 — Figure S2. Fluorescence of pollen tubes in self- and cross-pollinated pistils of tea plants at 48 h. “Top” and “Base” means the stigma and the base of the style of tea flower, respectively. Arrows indicate the pollen tubes with fluoresce. (TIF 4517 kb) [file 12864_2018_4674_MOESM2_ESM.tif]

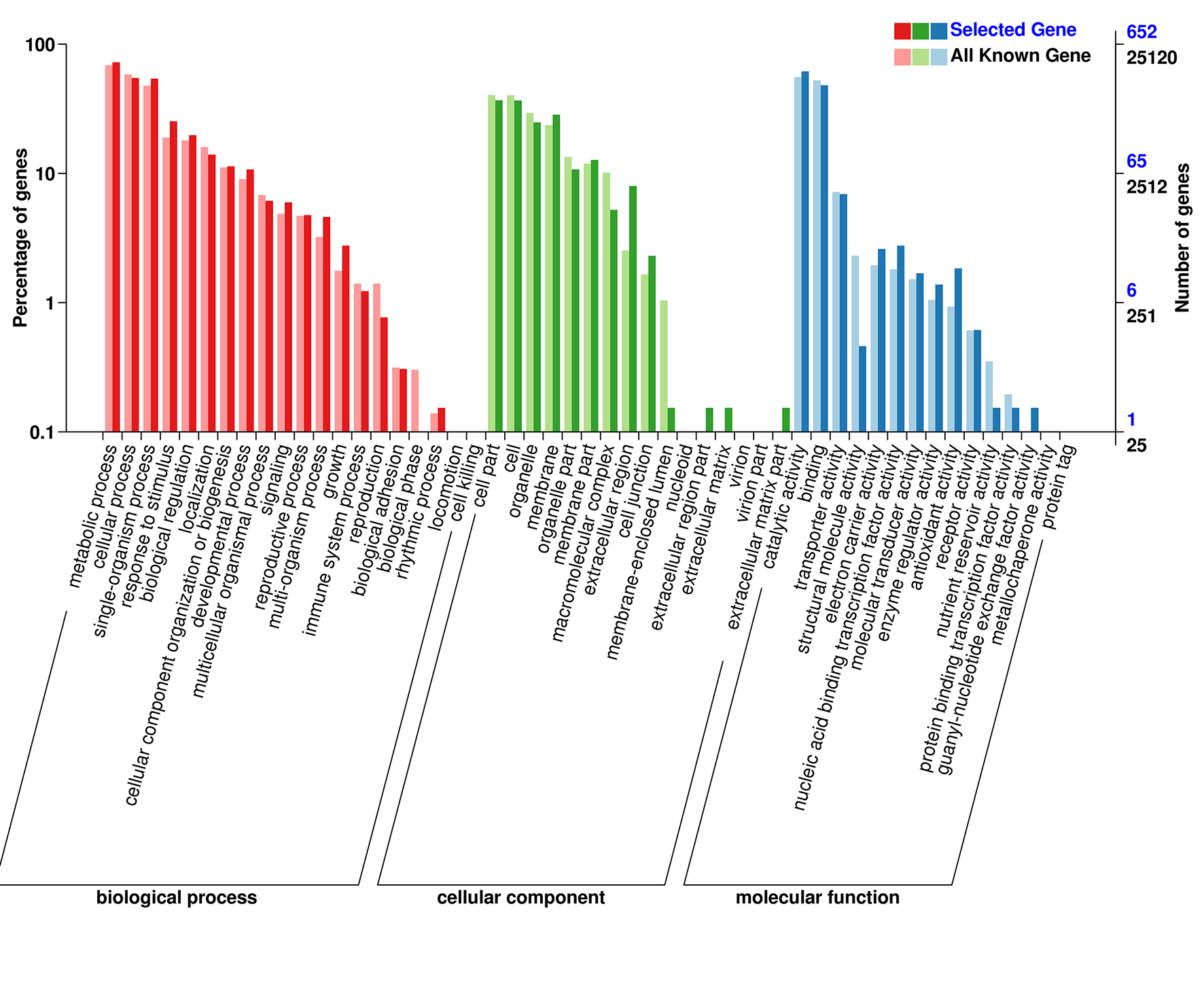

Supplement: Supplementary file 3 — Figure S3. Gene Ontology enrich analysis of DEGs between unpollinated and pollinated samples. (TIF 755 kb) [file 12864_2018_4674_MOESM3_ESM.tif]
